# Inhibition of the PI3K/Akt/mTOR Signaling Pathway in Diffuse Large B-Cell Lymphoma: Current Knowledge and Clinical Significance

**DOI:** 10.3390/molecules190914304

**Published:** 2014-09-11

**Authors:** Agata Majchrzak, Magdalena Witkowska, Piotr Smolewski

**Affiliations:** Department of Experimental Hematology, Medical University of Lodz, 93-510 Lodz, Poland

**Keywords:** diffuse large B-cell lymphoma, PI3K kinase, Akt kinase, mTOR kinase, inhibitor, treatment

## Abstract

Diffuse large B-cell lymphoma (DLBCL) is one of the most common non-Hodgkin lymphomas in adults. The disease is very heterogeneous in its presentation, that is DLBCL patients may differ from each other not only in regard to histology of tissue infiltration, clinical course or response to treatment, but also in respect to diversity in gene expression profiling. A growing body of knowledge on the biology of DLBCL, including abnormalities in intracellular signaling, has allowed the development of new treatment strategies, specifically directed against lymphoma cells. The phosphoinositide 3-kinase (PI3K)/protein kinase B (Akt)/mammalian target of rapamycin (mTOR) signaling pathway plays an important role in controlling proliferation and survival of tumor cells in various types of malignancies, including DLBCL, and therefore it may be a promising target for therapeutic intervention. Currently, novel anticancer drugs are undergoing assessment in different phases of clinical trials in aggressive lymphomas, with promising outcomes. In this review we present a state of art review on various classes of small molecule inhibitors selectively involving PI3K/Akt/mTOR pathway and their clinical potential in this disease.

## 1. Introduction

Diffuse large B-cell lymphoma (DLBCL) is the most common non-Hodgkin lymphoma (NHL) in adults and accounts for approximately 25% to 35% of newly diagnosed NHL cases annually. DLBCL is an aggressive type of lymphoma, very heterogeneous in clinical presentation, histology of tissue infiltration, prognosis and response to front line therapy [1]. The huge diversity of this disease was confirmed in gene expression profiling (GEP) revealing biologically and prognostically distinct subgroups, that stand for various stages of lymphocyte differentiation: germinal center B-cell like (GCB), activated B-cell like (ABC) and primary mediastinal B-cell lymphoma (PMBL) [2]. The GCB type overexpresses genes characteristic for normal germinal center B-cells, and the ABC subtype overexpresses genes of normal post germinal center B cells, that are ready to differentiate to plasma cells, whereas PMBL cells may arise from thymic B-cells [3]. GEP improved further understanding of the biology of DLBCL and gave a possibility to recognize genes and pathways that are pivotal to identify potentially important targets and therapies [4]. Despite the fact that DLBCL patients who receive standard chemotherapy have high chance to achieve partial or complete remission (PR or CR, respectively), there is still a significant group that is refractory to first line therapy or relapse after some time, therefore, new treatment approaches for this type of aggressive lymphoma are needed in order to create the chance to cure more patients.

## 2. Biology of the PI3K/Akt/mTOR Signaling Pathway

The phosphoinositide 3-kinase (PI3K)/protein kinase B (Akt)/mammalian target of rapamycin (mTOR) signaling pathway plays a crucial role in multiple cellular processes, including cell proliferation, angiogenesis, metabolism, differentiation and survival. It was already discovered that this pathway is often dysregulated in many cancers, including B-cell lymphomas [5].

The PI3Ks are intracellular lipid kinases that can be divided into three classes (named I-III) according to their substrate specificity and structural characteristics [6]. Class I is the most accurately characterized of all PI3Ks. It consists of two subunits that differ from each other in regard to receptors responsible for their activation. Class IA PI3Ks are activated by receptor tyrosine kinases (RTK) growth factor or G-protein-coupled receptors (GPCR) *via* interaction with Gβγ and also by Ras proteins, in this class kinases consist of a p85α, p85β or p55γ regulatory and a p110α, p110β, p110δ catalytic subunits isoforms. The genes that are responsible of encoding class IA regulatory subunits are *PIK3R1, PIK3R2* and *PIK3R3*, and for the catalytic p110(α, β, δ), *PIK3CA*, *PIK3CB* and *PIK3CD*, respectively. Class IB are activated by GPCRs and consist of just one catalytic p110γ and regulatory p101, p84 and p87PIKAP subunits [6,7,8]. Class II of PI3Ks are monomers and they exist in three isoforms: PI3KC2α, PI3KC2β, PI3KC2γ they lack regulatory subunits and have a single catalytic unit that interacts directly with phosphorylated adapter proteins [6]. Isoforms of class II have a similar sequence homology with class I p110 subunits and are encoded by *PIK3C2A*, *PIK3C2B*, *PIK3C2G* genes [7]. Class III of PI3Ks consist of catalytic hVps34 and a regulatory p150 subunit and are encoded by the PIK3C3 gene [6].

Although, all the PI3K classes are important, the one that plays the crucial role in oncogenesis is class I. The main function of PI3Ks is to phosphorylate the D3 position of phosphoinositide. The class I phosphorylate phosphatidylinositol-(4,5)-bisphosphate (PIP2) to generate phosphatidylinositol-(3,4,5)-trisphosphate (PIP3) the plasma membrane major secondary messenger and an essential effector of multiple downstream targets of the PI3K pathway [9,10]. This process could be reversed by tumor suppressor PTEN (phosphatase and tensin homologue) that converts PIP3 into inactive PIP2. Loss of PTEN is often connected with various different cancer types [6]. Among proteins that are initiated by PIP3 is an Akt, a serine-threonine kinase also known as the protein kinase B, one of the major oncogenic effectors of the PI3K/Akt pathway [6,11]. The PI3K/Akt pathway phosphorylates and activates Akt (pAkt), whose activated form stimulates multiple effectors that play an important role in cell survival and apoptosis [6]. One group of effectors that has a pivotal role in apoptosis inhibition are the Bcl-2 family proteins. Akt kinase negatively regulates the function or expression of several Bcl-2 homology domain 3 (BH3)—the only proteins that inactivate a prosurvival Bcl-2 protein family members [12]. The mTOR is a serine/threonine kinase laying downstream of the PI3K/Akt/mTOR pathway. 

Phosphorylated Akt activates mTOR complex 1 (mTORC1), causing increased mRNA translation, protein synthesis and cellular proliferation. Activation of a second mTOR complex (mTORC2), involved in regulation of the cytoskeleton, is probably an effect of Akt loop feedback [13]. The two mTOR complexes differ in structure. mTORC1 consist of Raptor and mLST8, while mTORC2 have mLST8 and Rictor. Initiated mTOR phosphorylates the ribosomal protein S6 kinase 1(S6K1) and the eukaryotic initiation factor 4E-binding protein 1 (4E-BP1), two proteins that are involved in the regulation of protein biosynthesis [14]. Activated S6K1 can inhibit the PI3K/Akt pathway by reducing expression of insulin receptor substrate 1 and 2, this inhibits the PI3K pathway [15].

## 3. PI3K/Akt/mTOR Signaling Pathway in DLBL

**Figure 1:**
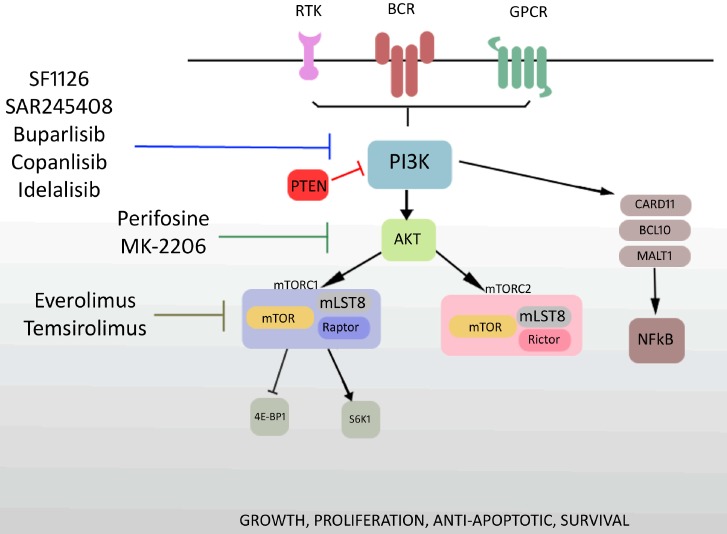
Schematic representation of the PI3K signaling pathway and sides of action PI3K/Akt/mTOR pathway inhibitors.

It has been already discovered that the PI3K/Akt/mTOR pathway is highly active in many malignancies, including lymphoid neoplasms. Activation of this pathway in DLBCL can result in gene mutations, loss of PTEN or constitutive activation of upstream regulatory pathways. The GCB subtype of DLBCL is defined by the loss of PTEN protein expression, while PTEN is expressed in the majority of ABC subtypes [16]. In the GCB the loss of PTEN correlates with PI3K/Akt/mTOR pathway activation, that as a result can be seen as phosphorylation of Akt. In contrast constitutive phosphorylation of Akt was not related with loss of PTEN in ABC DLBCL, that suggest that there is a different mechanism activating the PI3K/Akt/mTOR pathway in each DLBCL subtype [17]. The hallmark of the ABC subgroup of DLBCL is constitutive nuclear factor κB (NFкB) activation due to activation of the “CBM” signaling complex that consist of CARD11, BCL10 and MALT1, this complex may be constitutively stimulated by mutations of CARD11 and through chronic active B-cell receptor (BCR) signaling and downstream kinases including PI3K [18,19]. This signaling pathway is depicted in the Figure 1.

## 4. PI3K Inhibitors for DLBCL Treatment

As the PI3K/Akt/mTOR pathway plays a crucial role in cancer progression, including DLBCL, inhibition of this cascade became an obvious goal for novel treatment strategies. In this article we focus on some of the most attractive recent agents that are currently under investigation in clinical trials for DLBCL patients. All novel PI3K/Akt/mTOR pathway inhibitors mentioned in this article have been shown in Table 1.

BCR—B-cell receptor, 4E-BP1—4E binding protein, GPCR—G coupled protein receptor, PTEN—phosphatase and tensin homolog, RTK—receptor tyrosine kinase, S6K1—S6 kinase beta 1, PI3K—phosphoinositide 3-kinase, Akt—protein kinase B, mTOR—mammalian target of rapamycin, mTORC1—mammalian target of rapamycin complex1, mTORC2—mammalian target of rapamycin complex2, NFκB —nuclear factor κB.

**Table 1:** Selected clinical studies of targeted therapy in patients with diffuse large B-cell lymphoma.

Agent	Target	Type of Study	Indication	RR in DLBCL [%]	Reference
SF-1126	Class I—PI3K	Phase I	Advanced solid tumours B-cell malignancies	0	Mahadaven *et al.* [20]
SAR245408	Class I—PI3K	Phase I	Solid tumours NHL	25	Brown JR *et al.* [21]
Buparlisib	Class I—PI3K	Phase I	Solid tumours	0	Ando *et al.* [22]
Copanlisib	Class I—PI3K	Phase II	NHL	11	Dreyling *et al.* [23]
Idelalisib	PI3K (p110δ)	Phase I	NHL	0	Kahl *et al.* [24]
Perifosine	Akt	Phase II	CLL	ND	Friedman *et al.* [25]
MK-2206	Akt	Phase I	NHL	trial ongoing	Hiroshi *et al.* [26]
Everolimus	mTORC1	Phase II	NHL	30	Witzig *et al.* [27]
Temsirolimus	mTORC1	Phase II	NHL	28	Smith *et al.* [28]

PI3K—phosphoinositide 3-kinase, Akt—protein kinase B, DLBCL—diffuse large B-cell lymphoma, NHL—non-Hodgkin lymphoma, CLL—chronic lymphocytic leukemia, RR—response rate, mTORC1—mammalian target of rapamycin complex1, ND—no data.

### 4.1. SF1126

The SF1126 agent is an interesting small molecule prodrug containing the pan PI3K/mTOR inhibitor LY294002, that as a single agent it wasn’t a clinically viable drug, due to solubility problems and short half-life. The target for SF1126 are all class I PI3K isoforms and other members of PI3K/Akt/mTOR pathway including mTORC1, mTORC2. In a Phase I study by Mahadevan *et al.* [20] among patients with advanced solid tumours and B-cell malignancies SF1126 demonstrated safety, tolerability and efficacy mainly in the group with advanced solid tumors. Among a group of 39 patients, one patient with DLBCL diagnosis had more than 40% reduction in lymph node size after SF1126 administration. The most common adverse events (AEs) reported for SF1126 treated patients were oedema, alkaline phosphate increase, diarrhoea, weakness, hypoglycaemia, anaemia, urticarial/pruritus, hypokalaemia and hypersensitivity reaction. There were no grade 4 AE observed.

### 4.2. SAR245408

Another widely recently investigated selective class I PI3K inhibitor is SAR245408 (XL147), that inhibits phosphorylation of downstream targets of PI3K, including pAkt and pEBP1. SAR245408 was discovered by Exelixis, and is being co-developed by Sanofi-Aventis for use in endometrial cancer, breast cancer, glioblastoma, solid tumours and lymphoma [29]. A Phase I study included 25 patients—10 with refractory chronic lymphocytic leukemia (CLL) and 15 with relapsed or refractory lymphoma, including four with DLBCL diagnosis [21]. In the investigated group 80% of patients had advanced stages of the disease and almost half had bulky disease. Among DLBCL patients only one with transformed disease had progression during treatment, and progression free survival (PFS) was 18.4 months. SAR245408 is a promising single agent, that demonstrates a safe and tolerable profile with high clinical activity among patients with B cell malignancies, including DLBCL. The most common grade 3 or higher AEs observed in ≥10% of patients were neutropenia, diarrhea, anemia and hypotension.

### 4.3. Buparlisib

Another promising oral pan-PI3K inhibitor that selectively targets all four isoforms of class I (α, β, γ, δ) is buparlisib (NVP-BKM120). So far, it has shown efficacy both in *in vitro* and *in vivo* models [30]. In the study by Zang *et al.* [31] blocking the PI3K signaling pathway with buparlisib resulted in growth arrest and apoptosis of the DLBCL cell line. Moreover effective reduction of cell proliferation was observed on DLBCL cells obtained both from lymph nodes and from cavity fluid. The demonstrated effect is probably due to upregulation of BH-3 proteins including Puma and Bim with downregulation of Mcl-1 and Bcl-x_L_. A similar effect was observed in previously demonstrated data in solid tumors, mainly colorectal and breast cancer [22]. At the moment an international phase II clinical study with buparlisib in relapsed NHL including DLBCL is ongoing and is planned to be completed in 2014. The most common treatment-related AEs observed during buparlisib treatment were rash, increased transaminase levels, hyperglycemia and increased eosinophil count. 

### 4.4. Copanlisib

The novel intravenous single agent copanlisib (BAY 80-6946) is a highly selective, reversible PI3K inhibitor for class I PI3K α/β and δ isoforms, that was recently discovered and synthesized at Bayer HealthCare Pharmaceuticals [32]. Data published so far have shown toxicity similar to other class I inhibitors, with high response rates in patients with relapsed indolent NHL, small lymphocytic lymphoma (SLL)—67% and follicular lymphoma (FL)—40%, with 83% of responses in mantle cell lymphoma (MCL). In aggressive lymphomas the response rate was much lower, with 11% observed in the DLBCL group [23]. Grade 3 and 4 AEs were reported in 49% of patients, the most common being neutropenia, hypertension, hyperglycemia, diarrhea and easy fatigue (5%). 

### 4.5. Idelalisib

An example of a PI3K inhibitor with specific isoforms is idelalisib (formerly CAL-101 or GS-1101) nowadays widely investigated in a variety of malignant diseases. It is an oral, selective p110δ inhibitor that induces apoptosis in B-cell lines and primary B-cells from patients with B-cell malignancies including DLBCL [33]. In a phase I clinical study it was observed that idelalisib is an effective agent in relapsed indolent lymphoma both as a single agent and in combination with first line therapy. In work by Kahl *et al.* [24], among 55 refractory and relapsed NHL patients, who were orally administered idelalisib in monotherapy, nine had DLBCL diagnosis. Among side effects the most common were hematological laboratory abnormalities and liver enzymes elevations. Although the respective response rates were 69% and 55% for relapsed and refractory indolent NHL and 73% and 40% for MCL, respectively, no response was observed in the DLBCL group. Idelalisib was investigated as a part of combination therapy in patients with relapsed or refractory NHL [34]. OR rates and CR rates with the specific regimens were 85% and 27%, respectively, for idelalisib plus bendamustine, 72% and 19%, respectively, for idelalisib plus rituximab (RIT), and 71% and 43%, respectively, for idelalisib, bendamustine, and RIT. These results supported the need for the current, multicenter trials of idelalisib in various combinations with other drugs [34].

**Figure 2:**
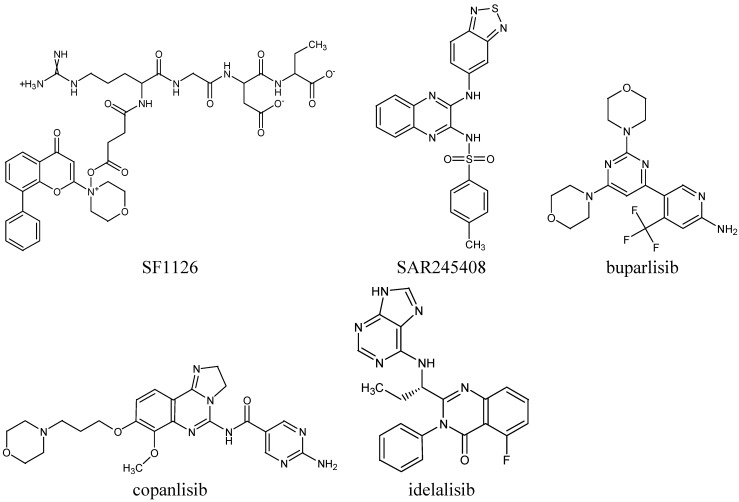
Chemical structures of phosphoinositide 3-kinase (PI3K) inhibitors with potential activity in diffuse large B-cell lymphoma.

Similar in structure to idelalisib but with a dual activity against both PI3K δ/γ isoforms is IPI-145 (INK-1197) [35]. This inhibitor shows safety and promising clinical activity against indolent NHL and MCL, with no efficacy in DLBCL patients [36]. Moreover, at 24 months, 69% of patients maintained their response, with 63% seeing no further progression of their cancer [37]. The chemical structures of the described PI3K kinase inhibitors are shown in Figure 2.

## 5. Akt Inhibitors as Agents Potentially Active in DLBCL

### 5.1. Perifosine

Perifosine (KRX-0401) is a first generation Akt inhibitor, that interferes with the interaction between the PH domain of Akt and PIP3, thereby preventing its translocation and activation [37]. It was discovered that perifosine is slightly active against B cell in indolent lymphomas, including CLL [25]. Due to the mild anticancer properties recently there are no clinical trials in patients with aggressive lymphoma. The drug was generally well tolerated, with very few grade 3 and 4 AEs. The most common drug-related toxicities included diarrhea, nausea, musculoskeletal pain, and easy fatigue.

### 5.2. MK-2206

**Figure 3:**
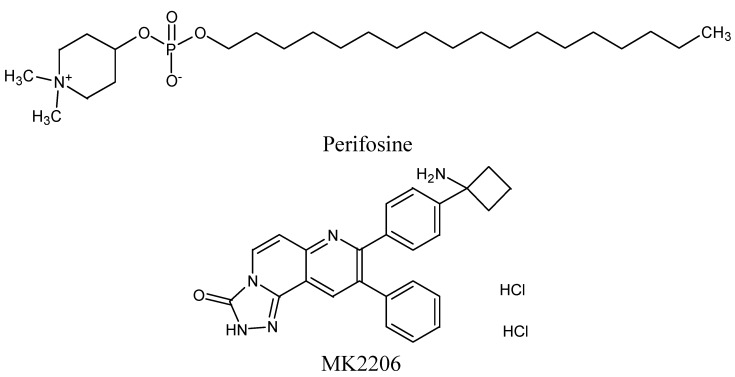
Chemical structures of protein kinase B (Akt) inhibitors with potential activity in diffuse large B-cell lymphoma.

Moreover, a highly selective second generation inhibitor of Akt, MK-2206, was discovered to be active in lymphoma patients [26]. It was already shown in preclinical trials to show strong cytotoxicity against cancer cells both in the investigated cell lines and in phase I trial in patients with solid tumors [38]. Nowadays there are clinical trials recruiting patients with aggressive lymphomas including DLBCL. The most common grade 3 and 4 AES was dehydration, hyperglycemia, rash and neutropenia. For the chemical structures of both Akt kinase inhibitors see Figure 3.

## 6. mTOR Inhibitors for Therapy of DLBCL

### 6.1. Everolimus

Another class of novel agents with anticancer activity demonstrated in preclinical trials, strongly associated with the PI3K/Akt/mTOR pathway, are rapamycin-like inhibitors. Everolimus (RAD-001) is an orally bioavailable mTOR inhibitor derivative of rapamycin (sirolimus) with antiproliferative effect connected mainly with mTORC1 inhibition, without involving mTORC2. Everolimus binds to FKBP-12, an intracellular protein, and directly inhibits mTORC1, reducing the activity of downstream effectors S6K1 and 4E-BP [15]. In a phase II clinical trial everolimus was administered in 77 patients with refractory and relapsed aggressive NHL at a 10 mg dose per day [27]. The overall response rate (ORR) among 47 DLBCL patients was 30%, with no CR observed and 14 PR. It was observed that everolimus, like other mTOR inhibitors, is well tolerated, with toxicity easily managed with dose reduction or interruption. In work by Xu *et al.* [39] the effect of RIT alone and in combination with rapamycin was evaluated in the DLBCL cell lines and 73 DLBCL patients. According to their results addition of rapamycin to RIT results in downregulation of the PI3K/Akt/mTOR pathway and this could be a promising option for chemotherapy-treated patients. The most common grade 3 and 4 drug related toxicities were neutropenia, anemia, and thrombocytopenia.

### 6.2. Temsirolimus

**Figure 4:**
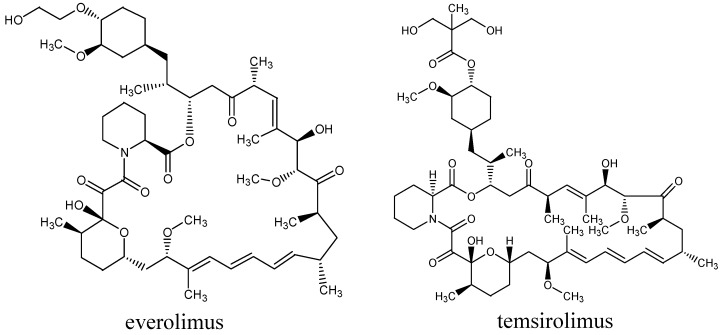
Chemical structures of mammalian target of rapamycin (mTOR) inhibitors with potential activity in diffuse large B-cell lymphoma.

Temsirolimus (CCI-779) is another intravenous mTOR inhibitor and a prodrug of rapamycin, that binds to the intracellular protein FKBP-12 and leads to cell cycle arrest in G_1_ phase; it also inhibits tumour angiogenesis by reducing the synthesis of vascular endothelial growth factor (VEGF) [40]. It was already demonstrated that temsirolimus has significant activity in B cell malignancies, including MCL, SLL or FL. In a phase II study by Smith *et al.* [28] in aggressive NHL, in the DLBCL group (*n* = 27) a single-agent temsirolimus dose of 25 mg weekly was investigated. Patients had relapsed disease after at least one prior regimen. In the DLBCL group overall response (OR) was 28.1% with 12.5% of CR. The most common AEs in this trial was thrombocytopenia. The chemical structures of both mTOR inhibitors are shown in Figure 4.

## 7. Conclusions

The lack of response of many DLBCL patients with refractory disease lead to a search for novel drugs that will be able to overcome cells resistance. So far, agents directly targeting PI3K/Akt/mTOR pathway, one of the most important in growth and survival signaling pathway in B cell malignancies, are hope for future treatment development both as a single agent and in combination with standard chemotherapeutics.
